# RALF signaling pathway activates MLO calcium channels to maintain pollen tube integrity

**DOI:** 10.1038/s41422-022-00754-3

**Published:** 2023-01-02

**Authors:** Qifei Gao, Chao Wang, Yasheng Xi, Qiaolin Shao, Congcong Hou, Legong Li, Sheng Luan

**Affiliations:** 1 Department of Plant and Microbial Biology, University of California at Berkeley, Berkeley, CA USA; 2 College of Life Sciences, Capital Normal University, Beijing, China

**Keywords:** Plant signalling, Calcium signalling

## Abstract

Pollen tube tip growth requires intricate Ca^2+^ signaling. Recent studies have also identified rapid alkalization factor (RALF)-family peptides and their receptors as critical components for pollen tube tip growth and integrity. The functional relationship of RALF and calcium signaling modules remains largely unclear. Here we report that disruption of RALF signaling pathway abolished the cytosolic Ca^2+^ gradient in the pollen tube, indicating that Ca^2+^ signaling is downstream of the RALF signaling pathway. We identified MILDEW RESISTANCE LOCUS O (MLO) family proteins MLO1, 5, 9, 15, as Ca^2+^ channels required for Ca^2+^ influx and pollen tube integrity. We further reconstituted the biochemical pathway in which signaling via RALF and RALF receptors activated MLO1/5/9/15 calcium channels. Together, we conclude that RALF peptides derived from pollen tube bind to their receptors to establish pollen tube Ca^2+^ gradient through activation of the MLO channels. Our finding has thus provided a mechanistic link between the RALF signaling pathway and Ca^2+^ signaling in controlling pollen tube integrity and growth.

## Introduction

Sperm cells in the flowering plants are immobile and rely on pollen tube for delivery to the ovule for fertilization. As soon as a pollen grain germinates on the stigma, a tip-focused cytosolic Ca^2+^ gradient is established in the pollen tube, which is essential for maintaining tube integrity, elongation, and guidance.^1^ Concerning the calcium channels involved in producing the calcium signals, studies have identified Cyclic nucleotide-gated channel 18 (CNGC18) as essential for both pollen tube growth^2^ and pollen tube guidance.^3^ A pair of CNGCs, CNGC7/8, are also essential for male fertility^4^ and have been shown to regulate CNGC18 activity in a Ca^2+^-calmodulin-dependent manner.^5^

In addition to calcium signals, RALF peptides derived from pollen tube are critical in maintaining pollen tube integrity and growth.^6^ In particular, RALF4/19 bind to the pollen tube receptor-like kinases, ANX1/2 (ANXUR1 and 2) and BUPS1/2 (BUDDHA’S PAPER SEAL 1 and 2),^7–9^ and their co-receptor LLG2/3 (LORELEI-like-GPI anchored protein 2 and 3)^10^ to activate downstream processes required for pollen tube integrity. One of such downstream components is the receptor-like cytoplasmic kinase (RLCK), MARIS, which acts as a positive regulator of pollen tube integrity/growth.^11^ The RALF family peptides also bind to the LEUCINE-RICH REPEAT EXTENSIN (LRX) family proteins that play a role in cell wall integrity and remodeling to sustain normal growth of pollen tube.^12,13^

Regarding functional relationship of RALF-RALF receptor pathway and calcium signaling, studies showed that cytosolic Ca^2+^ is elevated in response to several RALFs in the root.^14–16^ Furthermore, in the *anx1/2* double mutant, cytosolic Ca^2+^ spiking in the pollen tube is reduced.^17^ These results suggest that tip-focused calcium signal in the pollen tube may be downstream of RALF peptide signaling pathway.

MLO proteins are involved in plant powdery mildew susceptibility,^18,19^ root thigmomorphogenesis,^20^ and reproduction.^21,22^ Our latest study shows that MLOs function as Ca^2+^ channels unique to plants,^23^ indicating that MLOs in pollen tubes may also play important roles in Ca^2+^ signaling. Indeed, the tip-focused Ca^2+^ gradient of *mlo5mlo9* double mutant was much lower than that of WT plants,^22^ suggesting that MLOs are positive regulators of pollen tube Ca^2+^ signal.

Multiple factors and pathways described above appear to impinge on the regulation of pollen tube Ca^2+^ signals, but it remains unknown how they functionally interact to control pollen tube integrity/growth. Here we report that the disruption of any component of the RALF signaling pathway, including RALF4/19, ANX1/2, BUPS1/2, LLG2/3, or MARIS, abolished cytosolic Ca^2+^ gradient in the pollen tube, illustrating a functional link between the RALF-RLK-RLCK pathway and Ca^2+^ signaling. We further revealed that MLO1/5/9/15 are Ca^2+^ channels activated by ANX1/2-BUPS1/2-LLG2/3-MARIS pathway in response to RALF4/19.

## Results

### RALF4/19 are required and sufficient to trigger pollen Ca^2+^ elevation

**Fig. 1:**
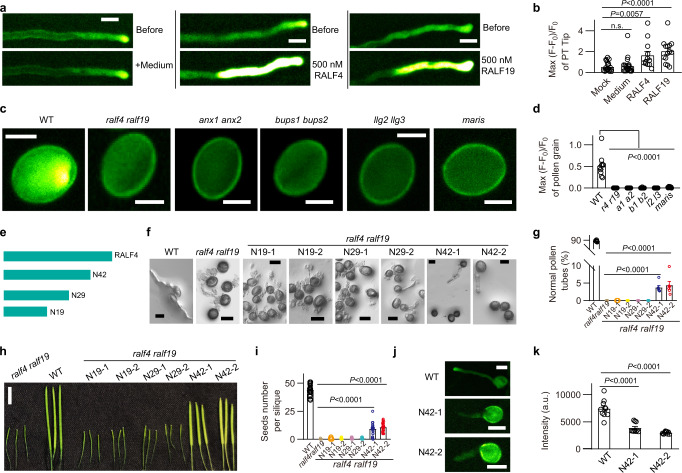
RALF4/19 pathway is required for generating pollen tube Ca^2+^ gradient. **a** RALF4 or RALF19 triggers a pollen tube Ca^2+^ elevation. Pollen germination medium was used as a control. Scale bars, 10 μm. **b** Statistical analysis of **a**. *n* = 20 pollen tubes for mock, *n* = 22 for medium, *n* = 12 for RALF4 and *n* = 14 for RALF19. **c** Pollen grain Ca^2+^ gradient is absent in the mutants of RALF4/19 signaling pathway. Scale bars, 10 μm. **d** Statistical analysis of **c**. *n* = 10 pollen grains. **e** The truncated versions of RALF4 mature peptide. **f** Complementation assay using various fragments of truncated RALF4. Scale bars, 20 μm. **g** Statistical analysis of **f**. *n* = 6 repeats, in each repeat about 100 pollen tubes were counted. **h**, **i** The siliques (**h**) and seed number per silique (**i**) of the complementation lines. Scale bars, 0.5 cm. *n* = 30 siliques. **j** Pollen tube Ca^2+^ gradient of N42 complementation lines. Scale bars, 20 μm. **k** Statistical analysis of **j**. *n* = 10 pollen tubes. Arbitrary units (a.u.) were used for the intensity. Error bars depict means ± SEM. All *P* values were determined by two-tailed Student’s *t*-test.

External RALFs induce cytosolic Ca^2+^ increase in root cells.^14–16^ We thus tested whether RALF4/19, which are essential for pollen tube integrity, can alter pollen tube Ca^2+^ signal. Using a transgenic plant expressing Ca^2+^ indicator (GCaMP6s) driven by the Ubiquitin 10 promoter,^24^ we observed a sharp Ca^2+^ increase in pollen tubes when applying 500 nM RALF4 or 19 to the medium (Fig. 1a, b; Supplementary information, Videos S1 and S2), suggesting that high concentrations of RALF4/19 trigger exaggerated pollen tube cytosolic Ca^2+^ elevation, which is detrimental to pollen tube growth. This result is consistent with the findings that exogenously applied RALF4/19 peptides reduce pollen tube growth,^12,13,16^ and that excess Ca^2+^ also inhibit pollen tube growth.^25^

We then tested whether RALF4/19 are required for pollen tube Ca^2+^ elevation. Because all pollen tubes burst in *ralf4ralf19* double mutant,^7^ hindering the effort to monitor its pollen tube Ca^2+^ changes, we turned to examine the Ca^2+^ signal during pollen germination phase. In the wild type pollen grains, after about 2 h incubation, we detected a Ca^2+^ spike in the aperture area of pollen grain before tube protrusion, followed by a tip-focused calcium signal in the elongating pollen tube (Fig. 1c, d; Supplementary information, Video S3). But in *ralf4ralf19* pollen grains, we did not detect any Ca^2+^ elevation before the pollen grain collapsed (Fig. 1c, d; Supplementary information, Video S4), indicating that RALF4/19 are also required for establishing a Ca^2+^ signal at the aperture before pollen tube formation. The findings that *ralf4ralf19* mutant failed to establish pollen tube Ca^2+^ gradient (Fig. 1c, d), and that applying exogenous RALF4/19 induced large Ca^2+^ elevation (Fig. 1a, b) which could inhibit pollen tube growth, suggested that pollen tube features a sophisticated mechanism to adjust the secretion of RALF4/19 to maintain an optimal level of Ca^2+^.

RALF4/19 initiate a signaling pathway that consists of receptor-like kinases ANX1/2, BUPS1/2, their co-receptors LLG2/3,^7,10^ and an RLCK, MARIS, to maintain pollen tube integrity and growth.^11^ As the mutants lacking any of these signaling components display pollen tube bursting phenotype, we utilized the same pollen germination assay described for *ralf* mutants to conduct calcium-imaging experiments. During the germination phase, no Ca^2+^ elevation was observed in *anx1/2*, *bups1/2*, *llg2/3* and *maris* mutants (Fig. 1c, d), suggesting that RALF4/19-ANX1/2-BUPS1/2-LLG2/3-MARIS signaling pathway is required for producing pollen Ca^2+^ signal.

To further investigate the role of RALF4/19 in eliciting calcium signals beyond pollen grain germination, we attempted to generate weaker alleles of *ralf4ralf19* mutant through complementation of the double mutant using partially functional fragments of the RALF4 peptide. It was reported previously that truncation of C-terminal region (containing four conserved cysteines) impaired but did not completely abolish RALF4 activity.^10^ We thus generated a series of transgenic plant lines harboring C-terminal truncated RALF4 driven by RALF4 promoter in the *ralf4ralf19* double mutant background (Supplementary information, Fig. S1). Based on the number of amino acids left in the N-terminal region, we named them as N19, N29 and N42 (Fig. 1e). The N19 and N29 lines, like the double mutant, showed 100% pollen tube bursting and did not generate any seeds (Fig. 1f‒i). Interestingly, pollen grains from N42-1 and N42-2 lines produced 3.74% and 4.37% intact pollen tubes, respectively (Fig. 1f, g), and about 10 seeds per silique (Fig. 1h, i), making it feasible to examine Ca^2+^ changes in pollen tubes. These two independent transgenic lines, N42-1 and N42-2 showed a reduced tip-focused Ca^2+^ level in the pollen tube as compared to the wild type (Fig. 1j, k), indicating that RALF4/19 are required for producing normal Ca^2+^ signals during pollen grain germination and pollen tube growth.

### MLO1, 5, 9 and 15 are Ca^2+^ channels activated by MARIS^R240C^

Signaling pathways initiated by RALFs induce cytosolic Ca^2+^ elevation in both root cells^14–16^ and pollen tubes (Fig. 1a, b), indicating that RALFs signaling pathway targets downstream components, such as Ca^2+^ permeable channels, responsible for Ca^2+^ influx.

**Fig. 2:**
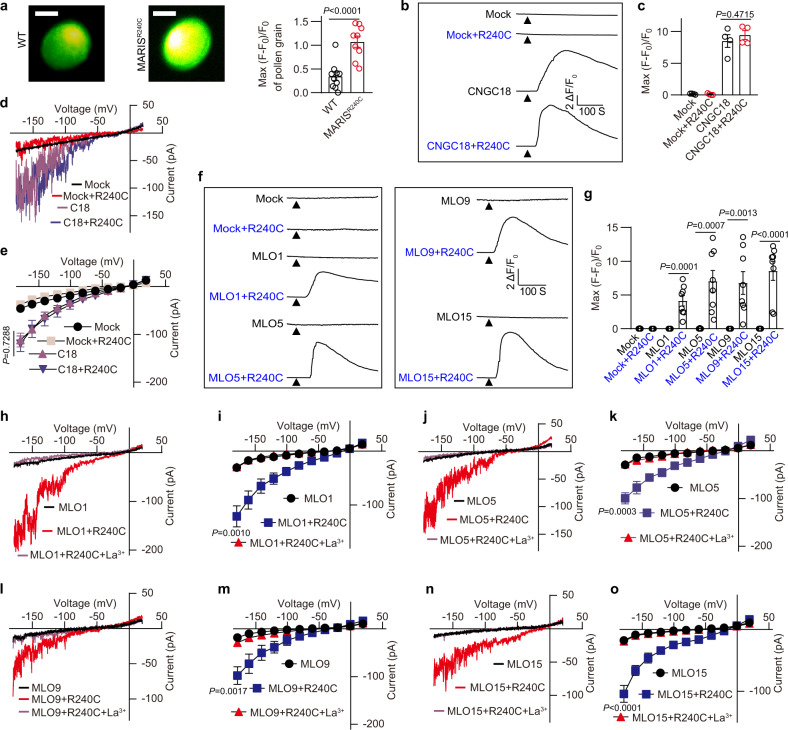
MLO1, 5, 9 and 15 are Ca^2+^ permeable channels activated by MARIS^R240C^. **a** Pollen grain Ca^2+^ gradient of WT and MARIS^R240C^-overexpressing plants. Scale bars, 10 μm. *n* = 10 pollen grains. **b**, **c** Representative cytosolic Ca^2+^ spiking curves (**b**) and statistical analysis of peak values (**c**) in COS7 cells expressing the CNGC18 or CNGC18 + MARIS^R240C^. *n* = 4 replicates, and ~60 cells were imaged in each duplicate. **d**, **e** Typical whole-cell recordings (**d**) and current-voltage curves (**e**) of inward currents in HEK293T cells expressing CNGC18 or CNGC18 + MARIS^R240C^. *n* = 6 cells. **f**, **g** Representative cytosolic Ca^2+^ spiking curves (**f**) and statistical analysis of peak values (**g**) in COS7 cells expressing the various MLOs and MARIS^R240C^. *n* = 8 replicates, and ~60 cells were imaged in each duplicate. **h**, **i** Typical whole-cell recordings (**h**) and average current-voltage curves (**i**) for MLO1 + MARIS^R240C^-mediated Ca^2+^ currents when expressed in HEK293T cells, and the inhibition of Ca^2+^ conductance by La^3+^ (100 μM). *n* = 6 cells. **j**‒**o** Similar analyses were conducted for other MLOs including MLO5 (**j**, **k**), MLO9 (**l**, **m**), MLO15 (**n**, **o**). *n* = 6 cells. Error bars depict means ± SEM. All *P* values were determined by two-tailed Student’s *t*-test.

To identify the downstream Ca^2+^ permeable channels, we turned our attention to the gain-of-function allele of MARIS, an RLCK downstream of the receptor kinases. As reported earlier, such RLCKs often link RLKs to calcium channels in response to peptide signals such as pathogen patterns.^26–28^ The dominant mutant of MARIS, MARIS^R240C^, has been shown to be constitutively active thereby suppressing the pollen tube bursting phenotype in *anx1anx2* double mutant lacking functional RALF receptors.^11^ In addition, overexpression of MARIS^R240C^ in the WT background inhibits pollen germination, indicating the importance of a balanced level of MARIS activity.^11^ If MARIS is an upstream activator of calcium channels, overexpression of MARIS^R240C^ may alter calcium levels in pollen tube. We overexpressed MARIS^R240C^ in WT plants harboring the Ca^2+^ indicator GCaMP6s and found that Ca^2+^ flooded almost the entire pollen grain during germination, instead of forming a polarized Ca^2+^ signal at the aperture (Fig. 2a; Supplementary information, Video S5). This result suggests that MARIS^R240C^, like addition of RALF peptides (Fig. 1a, b), is a robust activator for pollen Ca^2+^ signal and may activate Ca^2+^ channels directly.

One of the potential Ca^2+^ channel candidates targeted by MARIS^R240C^ is CNGC18 that has been shown to play a role in pollen tube integrity.^2^ However, CNGC18 is active when expressed alone,^3,5,29^ and its activity was not further enhanced by co-expressing with MARIS^R240C^ (Fig. 2b‒e), indicating that CNGC18 may not be the functional target of MARIS^R240C^.

Another family of potential MARIS targets could be MLOs as some MLO members facilitate calcium influx when expressed in COS7 and HEK293T cells.^23^ However, none of the pollen-expressed MLO1/5/9/15 mediated Ca^2+^ entry when expressed in COS7 cells^23^ (Fig. 2f, g), suggesting that the four MLOs may require specific regulators to become active.^26,30,31^ Indeed, when *At*MLO1, 5, 9 or 15 was co-expressed with MARIS^R240C^, each of them mediated Ca^2+^ influx (Fig. 2f, g). To confirm the Ca^2+^ imaging results, we used patch-clamp to directly measure transport activity of *At*MLO1, 5, 9 and 15 and recorded large inward currents when they were co-expressed with MARIS^R240C^ in the HEK293T cells (Fig. 2h‒o). Furthermore, the typical Ca^2+^ channel blockers lanthanum (La^3+^) inhibited the *At*MLO1, 5, 9 or 15-mediated inward currents (Fig. 2h‒o). These results indicate that *At*MLO1, 5, 9 and 15 function as MARIS^R240C^-activated Ca^2+^-permeable channels.

### MLO1, 5, 9 and 15 are required for pollen tube integrity and directly interact with MARIS

Ca^2+^ imaging and electrophysiological experiments showed that MARIS^R240C^ activated MLO1, 5, 9 and 15 (Fig. 2f‒o). If MLOs are functional targets of MARIS^R240C^, they should also be required for pollen tube integrity. But *mlo5mlo9* double mutant, unlike RALF signaling mutants, did not show pollen tube bursting phenotype.^22^ We suspected that other MLOs, including MLO1 and MLO15, are also expressed in the pollen tube, which may lead to more complex functional redundancy. Thus, we attempted to mutate the four pollen tube MLOs and examine whether the higher order mutants had pollen tube bursting phenotype. Since *MLO1*, *5* and *9* are located at different chromosomes, we first generated a *mlo1mlo5mlo9* triple mutant by crossing T-DNA insertional single mutants. In the triple mutant background, we deleted MLO15 using the CRISPR procedure. But we failed to isolate a quadruple mutant after screening a large number of plants, possibly due to infertility resulting from mutating all four MLOs. We then focused on the *mlo1mlo5mlo9* triple mutant for phenotyping.

**Fig. 3:**
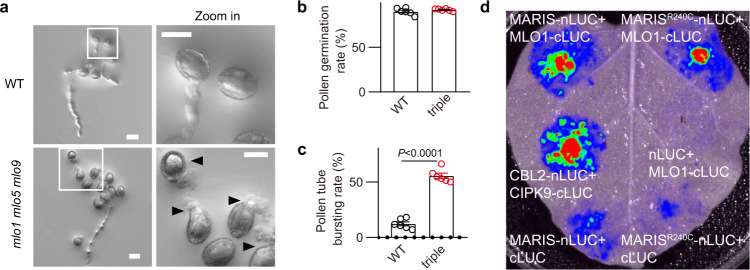
MLO1, 5, 9 and 15 are required for pollen tube integrity. **a** The pollen germination of WT and *mlo1mlo5mlo9* triple mutant. The white rectangle indicated the area magnified in the right panel. Arrowheads indicate the bursting pollen tubes after germination. Scale bars, 20 μm. **b**, **c** Pollen germination rate (**b**) and pollen tube bursting rate (**c**) of WT and *mlo1mlo5mlo9* triple mutant. *n* = 6 repeats, and in each repeat about 100 pollen tubes were counted. **d** Firefly luciferase (LUC) complementation imaging assay. *N. benthamiana* leaves were co-infiltrated with agrobacterial strains containing different pairs of constructs. Error bars depict means ± SEM. All *P* values were determined by two-tailed Student’s *t*-test.

Although male transmission efficiency of the *mlo5mlo9* double mutant and *mlo1mlo5mlo9* triple mutant were reduced (Supplementary information, Table S1),^22^ the triple mutant showed a more severe defect (Supplementary information, Table S1), indicating a functional redundancy of MLO1, 5 and 9 in male fertility. More importantly, we found that *mlo1mlo5mlo9* triple mutant showed a new phenotype: pollen tube bursting rate was significantly higher than that in the WT (Fig. 3a‒c). This result indicated that MLO1, 5, 9, and perhaps MLO15, like RALF pathway components, are required for pollen tube integrity.

We then examined the link between MARIS and MLO by a protein‒protein interaction assay. As MLO1/5/9/15 are highly redundant, we used MLO1 and MARIS to carry out a split‐luciferase complementation (LUC) assay in *Nicotiana benthamiana*. The N-terminal portion (nLUC) of firefly luciferase was fused to MARIS/MARIS^R240C^ and the C-terminal portion (cLUC) was fused to MLO1. As a positive control, CBL2 was fused to nLUC and CIPK9 was fused with cLUC.^32^ Strong fluorescence signals were detected in the leaf cells expressing MARIS/MARIS^R240C^-nLUC and MLO1‐cLUC, as well as in the cells expressing positive control, whereas no discernible signals were observed in the cells expressing negative controls (Fig. 3d), suggesting that MARIS and MARIS^R240C^ interacted directly with MLO1.

### Reconstitution of the RALF4/19 signaling pathway targeting MLO family Ca^2+^ channels

Unlike MARIS^R240C^, wild type MARIS did not activate *At*MLO1/5/9/15-mediated Ca^2+^ influx when co-expressed in COS7 cells (Supplementary information, Fig. S2a, b), suggesting that MARIS may require upstream activators. Since MARIS are downstream of the RLKs and co-receptors for RALFs, we then co-expressed these components with MARIS and MLO5 to test if they indeed form a linear pathway. The receptors or MARIS alone, or combination of receptors and MARIS failed to activate MLO5 (Supplementary information, Fig. S3), showing that the ANX1/2-BUPS1/2-LLG2/3-MARIS are not active in this expression system.

**Fig. 4:**
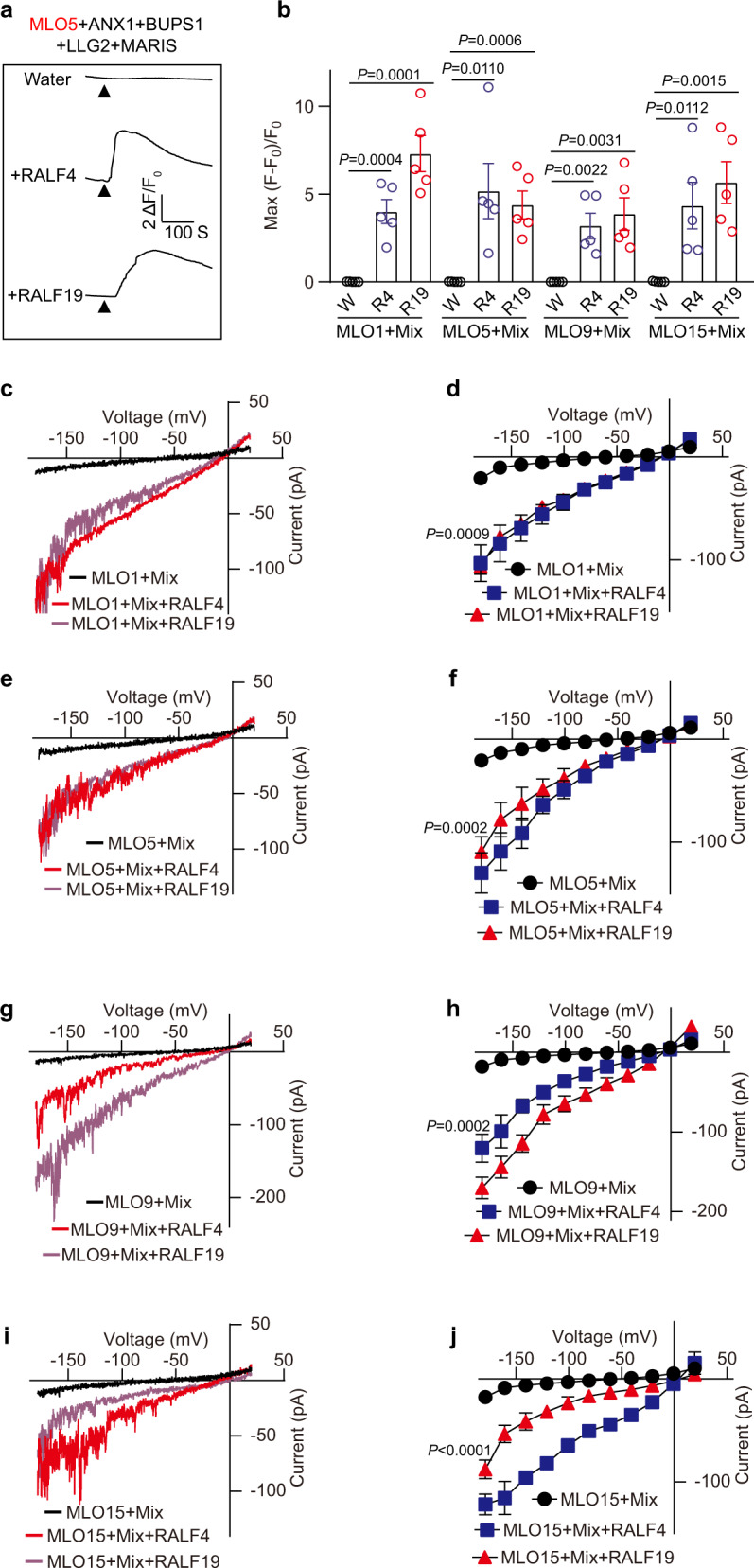
MLO1, 5, 9 and 15 are activated by RALF4/19 signaling pathway. **a**, **b** Representative cytosolic Ca^2+^ spiking curves (**a**) and statistical analysis of peak values (**b**) in COS7 cells expressing the MLO1/5/9/15 and RALF4/19 signaling components with 500 nM RALF4 or RALF19. *n* = 5 replicates, and ~60 cells were imaged in each duplicate. **c**, **d** Typical whole-cell recordings (**c**) and average current-voltage curves (**d**) for HEK293T cells co-expressing MLO1 and RALF4/19 signaling components (Mix) with 500 nM RALF4 or RALF19. W denotes water control. *n* = 6 cells. **e**–**j** Similar analyses were conducted for other MLOs including MLO5 (**e**, **f**), MLO9 (**g**, **h**), MLO15 (**i**, **j**). *n* = 6 cells. Error bars depict means ± SEM. All *P* values were determined by two-tailed Student’s *t-*test.

As the ANX1/2-BUPS1/2-LLG2/3-MARIS signaling pathway need to be activated by RALF4/19 peptides in pollen tube, we ventured to apply 500 nM RALF4 or RALF19 in the medium bathing COS7 cells for Ca^2+^ imaging assay. Strikingly, addition of 500 nM RALF4 or RALF19 strongly activated calcium elevation in cells expressing ANX1/2-BUPS1/2-LLG2/3-MARIS and MLO5, MLO1, 9, or 15 (Fig. 4a, b; Supplementary information, Fig. S4). This Ca^2+^ imaging result was also confirmed by our patch-clamp recording in HEK293T cells (Fig. 4c‒j).

### The BUPS1 receptor kinase phosphorylates MARIS

**Fig. 5:**
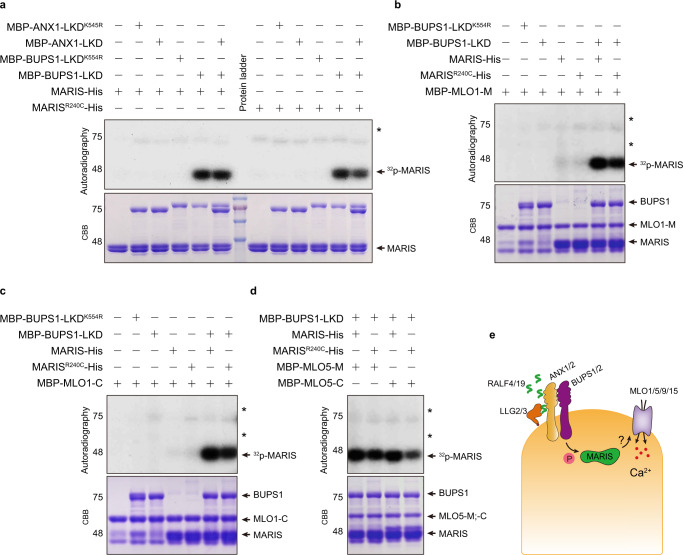
BUPS1 phosphorylates MARIS. **a** BUPS1, but not ANX1, phosphorylates MARIS and MARIS^R240C^ in vitro. Kinase-dead versions of ANX1 (MBP-ANX1-LKD^K545R^) and BUPS1 (MBP-BUPS1-LKD^K554R^) were used as negative controls. **b**–**d** BUPS1, MARIS or MARIS^R240C^ did not phosphorylate MLO1 or MLO5. MBP-tagged cytosolic domains of MLO1 (**b**, **c**) and MLO5 (**d**) were incubated with MARIS, MARIS^R240C^ or BUPS1. MLO-M indicates the cytosolic loop between the third and fourth transmembrane domains. MLO-C indicates the C-terminal cytosolic domain. **e** A model showing the activation of MLO1/5/9/15 by the RALF4/19 signaling pathway. Pollen-derived RALF4 and 19 bind to their receptors that in turn activate the RLCK, MARIS, leading to activation of MLO1, 5, 9 and 15 to mediate Ca^2+^ influx.

The signaling pathway of ligand-RLK-RLCK is usually transduced by a phosphorylation cascade. For example, in the pattern-triggered immune response pathway, the pattern peptide flg22 binds to the plant RLK receptor FLS2 that in turn associates with another RLK, BAK1, which phosphorylates and activates a RLCK, BIK1, to transduce the immune signal by phosphorylating downstream targets including calcium channels.^26,33^ In the RALF4/19-RLK-MARIS pathway, genetic analyses identified two pairs of RLKs, ANX1/2 and BUPS1/2, to serve as receptors for RALF4/19. However, it remains unknown if any of these RLKs directly phosphorylate MARIS and if MARIS in turn phosphorylates targets such as MLOs. As a step to biochemically connect the pathway components, we expressed and purified recombinant proteins, including kinase domains of ANX1 and BUPS1, MARIS, MARIS^R240C^, and soluble portions of MLOs, to perform in vitro kinase assays. When ANX1/BUPS1 kinase domains fused with the MBP tag were incubated with the His-tagged MARIS/MAR^R240C^, neither ANX1 nor BUPS1 has auto-phosphorylation activity. Interestingly, BUPS1, but not ANX1, strongly phosphorylated MARIS and MARIS^R240C^ (Fig. 5a).

Because MARIS/ MARIS^R240C^ physically interacted with MLOs and activated their channel activities (Figs. 2f‒o and 3d), we hypothesized that they may phosphorylate MLOs. To test this idea, the cytosolic regions of MLO1 and MLO5, including the large middle loop domain and the C-terminal tails, were expressed with MBP tags and incubated with His-tagged MARIS/MARIS^R240C^ in the kinase reaction. However, we did not detect any phosphorylation signal, i.e., neither auto-phosphorylation activity of MARIS and MARIS^R240C^ nor trans-phosphorylation of MLOs was observed (Fig. 5b‒d). This result, together with a previous report,^34^ showed that MARIS, although there is no reason to believe that it is not a functional kinase, may require some specific modification in order to become active. Such modification may include phosphorylation by the upstream kinases such as BUPS1/2. We then pre-incubated MARIS/MARIS^R240C^ with BUPS1, followed by adding MLOs to the reaction, but we again failed to detect any phosphorylation of MLOs (Fig. 5b‒d), indicating that although MARIS was phosphorylated by BUPS1, neither BUPS1 nor phosphorylated-MARIS can phosphorylate MLOs. The mechanism underlying activation of MLOs by MARIS remains unknown and may require future experiments using native tissues such as pollen tube to examine the biochemical relationship of these components.

## Discussion

In this study, we showed that RALF4/19 initiated the ANX1/2-BUPS1/2-LLG2/3-MARIS cascade that in turn activated *At*MLO1, 5, 9 and 15 (Fig. 5e) to enable tip-focused Ca^2+^ influx and maintain pollen tube integrity, providing evidence that MLOs are Ca^2+^ channels downstream of RALF peptide signaling pathway.

Like RALF peptides, Ca^2+^ signal is also essential for pollen tube integrity and the dialog between male (pollen tube) and female gametophytes (ovule) during pollen tube guidance and reception.^3,35–37^ Although it is well-established that RALF signaling plays a critical role in plant growth, immunity, and reproduction,^7,10,12,13,15,38–41^ little is known about how RALFs generate Ca^2+^ signal in any of these processes. Our study on pollen tube-synergid interaction^23^ suggest that MLO family Ca^2+^ channels are downstream of RALF peptide signaling, which shed light on the mechanistic link between RALF signaling and the encoding mechanisms of calcium signatures. Considering the functional diversity of MLO family proteins in plants,^42^ it is possible that MLOs also function downstream of other RALF-RLK signaling events beyond plant reproduction.

Although we found that MLOs are calcium channels downstream of the RALF-RLK-MARIS pathway, it remains unknown if other channels, such as CNGC18, may also play a role in the same process. Another question is how calcium signaling is connected with cell wall integrity: while the mutants lacking any of the components in the RLKs-MARIS-MLOs pathway are defective in pollen tube integrity and calcium signaling, it remains unclear if calcium signaling is directly linked to cell wall integrity. In this regard, signaling processes involving Rho-like GTPase from Plant (ROP) may be particularly relevant. In pollen tubes, ROPs promotes exocytosis that facilitates secretion of RALF4/19 for mechanical signal amplification and cell wall rigidification.^40^ In leaf pavement cells, another CrRLK1L, FERONIA, binds demethylesterified pectin to activate the intracellular ROP GTPase signaling pathway, connecting mechanical stress to cell morphogenesis in plants.^43,44^ It appears that RLKs-MARIS-MLOs may be a more directly connected pathway and ROP signaling may be associated through a crosstalk mechanism.

Another important finding in this study is that BUPS1, but not ANX1, phosphorylates MARIS and MARIS^R240C^ (Fig. 5a), bridging a biochemical gap between the pollen tube RLKs and the RLCK. Indeed, previous studies provide strong genetic evidence connecting RALF4/19, RLKs and MARIS in regulation of pollen tube integrity.^7,10–12^ However, biochemical relationship of RLKs and MARIS remains unclear. In fact, the kinase activity of many of the RLKs in the *Catharanthus roseus* RLK1-like (CrRLK1L) subfamily, including FERONIA, ANX1/2 and BUPS1/2, have not been studied despite the works showing their ligand binding properties of the extracellular domains.^45,46^ In our studies, we found it very challenging to successfully express and purify these kinase domains in heterologous systems. In the general framework of RLK signaling, the finding of MARIS phosphorylation by BUPS1 suggests that ANX1/2 may play a primary role in ligand perception while BUPS1/2 may function predominantly in transducing the ligand signal by phosphorylating and activating MARIS, an RLCK. In an attempt to connect MARIS to its potential substrates MLOs, however, we were not able to detect the phosphorylation of MLO1/5/9/15 by MARIS or MARIS^R240C^ (Fig. 5b‒d), although the constitutively active form of MARIS, MARIS^R240C^, activates the Ca^2+^ channel activities of MLO1/5/9/15 in the mammalian cell systems (Fig. 2f‒o). Purification of fully active MARIS may require use of native tissues such as pollen tubes, which should be achieved by future experiments.

## Materials and Methods

### Plant materials and growth conditions

Seeds were sterilized with 10% (v/v) bleach and sown on agar plates containing 1/2 MS medium (1/2 MS, 0.8% (w/v) Phyto agar, and 1% (w/v) sucrose, pH adjusted to 5.8 with KOH). Plates were incubated at 4 °C for 3 days for stratification and then seeds were transferred to the soil pots in 22 °C growth room with a 16-h light/8-h dark cycle (100 μmol m^−2^ s^−1^). The seeds for *mlo1* (CS881485), *mlo5* (SALK_118934C) and *mlo9* (SALK_073198) were purchased from *Arabidopsis* Biological Resource Center. The *ralf4ralf19, llg2llg3, bups1bups2* mutants were generated by CRISPR as reported.^7,10^ The plant harboring Ca^2+^ indicator GCaMP6s is from the Wolf B. Frommer’s lab,^24^ and *anx1anx2*, *maris* mutants are kindly provided by Ueli Grossniklaus and Aurélien Boisson-Derniera.^11,17^

### Transgenic plants

The coding DNA sequence (CDS) of MARIS was PCR-amplified from Columbia-0 (Col-0) cDNA and fused to the *Ubquitin10* promoter region amplified from Col-0 genomic DNA in the pCAMBIA 1305 vector. Then MARIS^R240C^ was generated by PCR-based site-directed mutagenesis. The binary construct was transformed into *Arabidopsis thaliana* (Col-0) plants via *Agrobacterium tumefaciens* GV3101 using the floral dip method.^47^ Transgenic plants were selected on 1/2 MS plates containing 35 mg/L hygromycin.

### Mammalian cell culture, vector construction, and transfection

The CDS of GCaMP6s was amplified from HBT-GCaMP6-HA^48^ and cloned into a dual-promoter vector, pBudCE4.1 (Invitrogen), with each CDS for MLOs or ANX1/BUPS1/LLG2/MARIS for co-expression in HEK293T or COS7 cell.

Mammalian cells were cultured in Dulbecco’s Modified Eagle’s Medium (DMEM) supplemented with 10% fetal bovine serum in a 5% CO_2_ incubator at 37 °C with controlled moisture. HEK293T or COS7 cells were transfected using Lipofectamine™ 3000 Transfection Reagent Kit (Invitrogen). Plasmids for transfection were extracted from *E. coli* (DH5α) using QIAGEN Plasmid Mini Kit (Qiagen), and 2 μg plasmid DNA was added into each well of 6-well plates (Nunc) containing the cells (70%–80% confluent). To confirm that the cells were successfully transfected, green and/or red fluorescent signals were examined using an inverted fluorescence microscope (Zeiss AxioObserver Z1 Inverted Microscope) before patch clamp and Ca^2+^ imaging experiments were performed 48 h after transfection.

### Whole-cell patch-clamp recording

The whole-cell patch-clamp experiments were performed using an Axopatch-200B patch-clamp setup (Axon Instruments, CA, USA) with a Digitata1550 digitizer (Axon Instruments, CA, USA) as previously described.^3,29^ Clampex10.7 software (Axon Instruments, CA, USA) was used for data acquisition and Clampfit 10.7 was used for data analysis.

To record Ca^2+^ currents across the plasma membrane of HEK293T cells, the standard bath solution contained 140 mM *N*-Methyl-d-Glucamine (NMDG)-Cl, 10 mM CaCl_2_, 10 mM glucose, and 10 mM HEPES, adjusted to pH 7.2 with Ca(OH)_2_. The standard pipette solution contained 140 mM Cs-glutamate, 6.7 mM EGTA, 3.35 mM CaCl_2_, and 10 mM HEPES, adjusted to pH 7.2 with CsOH. Free Ca^2+^ in the pipette solution was 175 nM, as calculated using the Webmaxc Standard (web.stanford.edu/∼cpatton/webmaxc/webmaxcS.htm). A ramp voltage protocol of 2-s-duration from ‒180 mV to +30 mV (holding potential 0 mV) was applied 1 min after accessing to a whole-cell configuration, and currents were recorded every 20 s for 5 repeats in total for each cell. The 5 current traces were used for statistical analysis for average current-voltage curves.

### Single-cell Ca^2+^ imaging in mammalian cells

HEK293T or COS7 cells expressing GCaMP6s and various combinations of candidate channel proteins were monitored by a Zeiss AxioObserver Z1 Inverted Microscope (Ivision 4.5 software) using a 20× objective as previously reported.^5^ The interval of data acquisition was 2 s. The standard solution for Ca^2+^ imaging contained 120 mM NaCl, 3 mM KCl, 1 mM MgCl_2_, 1.2 mM NaHCO_3_, 10 mM Glucose, 10 mM HEPES, pH 7.5. About 60 s after initiation of imaging procedure, the bath was perfused using a peristaltic pump with the standard solution supplemented with 10 mM Ca^2+^ and/or RALFs to elicit Ca^2+^ entry through active channels.

### Pollen tube Ca^2+^ imaging

For RALF-induced pollen tube [Ca^2+^]_cyt_ elevation experiment, pollen grains were germinated in the pollen germination medium (PGM) containing 18% sucrose, 0.01% boric acid, 1 mM MgSO_4_, 1 mM CaCl_2_, 1 mM Ca(NO_3_)_2_ and 0.5% agarose, pH 7.0.^49^ After 6-h incubation at 22 °C and 100% relative humidity, pollen tubes expressing GCaMP6s were monitored by a Zeiss AxioObserver Z1 Inverted Microscope (Ivision 4.5 software) using a 20× objective, and various RALFs were added to the PGM as indicated.

For pollen germination imaging, pollen grains were spread on the surface of PGM, and then monitored immediately using a Zeiss AxioObserver Z1 Inverted Microscope (Ivision 4.5 software) using a 20× objective.

### Peptide purification

The pFastBac constructs expressing RALF4, RALF19 and LRX8 were kind gifts from Julia Santiago of University of Lausanne, and RALF4/19 peptides were purified as reported.^13^

High Five cells were infected with virus with a multiplicity of infection (MOI) of 3 and incubated for 1 day at 28 °C and 2 day at 22 °C at 110 rpm on an orbital shaker. The secreted peptides were purified from the supernatant by Ni^2+^ column (Ni-NTA, Qiagen), and incubated with TEV protease (New England Biolabs) to remove the tags. Peptides were further purified by size-exclusion chromatography on a Superdex 200 increase 10/300 GL column (GE Healthcare), equilibrated in 20 mM sodium citrate, pH 5.0, 150 mM NaCl. The peptides were diluted with sterile pure water before use.

### Transient expression in *N. benthamiana* leaves

*Agrobacterium tumefaciens* GV3101 carrying proper constructs were grown in Luria-Bertani medium overnight until optical density (OD) at 600 nm was about 1.0. *A. tumefaciens* cells were collected and suspended in infiltration buffer (10 mM MgCl_2_, 10 mM MES-KOH, pH 6.0, and 200 μM acetosyringone) at a final OD600 = 0.5. For coexpression of two proteins, same amounts of *A. tumefaciens* cells carrying proper constructs (final OD600 = 0.5) and *A. tumefaciens* carrying p19 helper plasmid (final OD600 = 0.3) were mixed. The cells were incubated at room temperature for 2 h before infiltration. Leaves were observed 36‒48 h after infiltration under a Zeiss 710 confocal microscope. The leaves expressing split-luciferase complementation constructs were sprayed with 1 mM d-luciferin (Neta Scientific) and the luciferase activities that indicate protein‒protein interactions were detected by a BioRad CCD imaging system.

### In vitro phosphorylation assay

ANX1, BUPS1 kinase domains and MLO cytosolic regions including the middle loop domain or the C-tails were expressed using pMAL-c2x vector with C-terminal MBP fusion. MARIS and MARIS^R240C^ were expressed as His-tag proteins using pET28a. Proteins were expressed in Rosetta (DE3) and purified using standard procedures. Eluted proteins were desalted with a buffer containing 20 mM Tris-HCl, pH7.5 and 1 mM DTT before use. For the kinase assay, 2 or 3 μg of each protein was incubated in the kinase reaction buffer containing 20 mM Tris-HCl, pH 7.5, 10 mM MgCl_2_, 10 mM MnCl_2_, 1 mM DTT, 10 µM adenosine triphosphate (ATP) and 5 µCi γ-^32^P-labeled ATP at 28 °C for 1 h, and the reaction was terminated in the SDS-PAGE loading buffer at 65 °C for 5 min, followed by 12% SDS-PAGE, autoradiography and Coomassie Brilliant Blue (CBB) staining.

### Image processing and data analysis

Using the imageJ (1.51j8 version) software, GCaMP6s signals were analyzed overtime at several regions of interest. To calculate the fractional fluorescence changes (ΔF/F), the equation ΔF/F = (F − F_0_)/F_0_ was used, where F_0_ denotes the average baseline fluorescence determined by the average of F over the first 10 frames of the recording before the treatment.

Microsoft Excel in office 365 and GraphPad Prism 7.0 were used for calculation and statistical analysis of the data; Adobe Illustrator CC 2019 was used for image assembly; Clampfit 10.7 was used to analyze and process data from electrophysiological experiments.

## Supplementary information

VS1

VS2

VS3

VS4

VS5

Fig. S1

figS2

figS3

figS4

Table S1

video description
