# Supplementary material for: RALF signaling pathway activates MLO calcium channels to maintain pollen tube integrity
Source: Cell Res. 2023 Jan 2;33(1):71–9. doi: 10.1038/s41422-022-00754-3 (PMC9810639; doi:10.1038/s41422-022-00754-3)
Supplement: Supplementary file 6 — Fig. S1 [file 41422_2022_754_MOESM6_ESM.pdf]

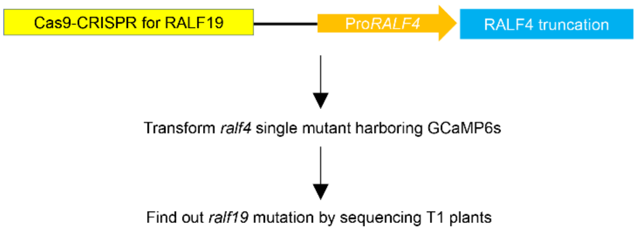

Supplementary information, Fig.1 The workflow for generating *ralf4 ralf19* complementing lines. A pCAMBIA 1300 vector containing two units, one for mutating *RALF19* by the CRISPR technique and the other for expressing RALF4 truncated versions driven by the *RALF4* promoter, was used to transform *ralf4* single mutant harboring the Ca^{2+} indicator GCaMP6s. The T1 plants were genotyped by DNA sequencing to identify lines containing both *ralf19* mutations and truncated RALF4.
