# Supplementary material for: RALF signaling pathway activates MLO calcium channels to maintain pollen tube integrity
Source: Cell Res. 2023 Jan 2;33(1):71–9. doi: 10.1038/s41422-022-00754-3 (PMC9810639; doi:10.1038/s41422-022-00754-3)
Supplement: Supplementary file 7 — figS2 [file 41422_2022_754_MOESM7_ESM.pdf]

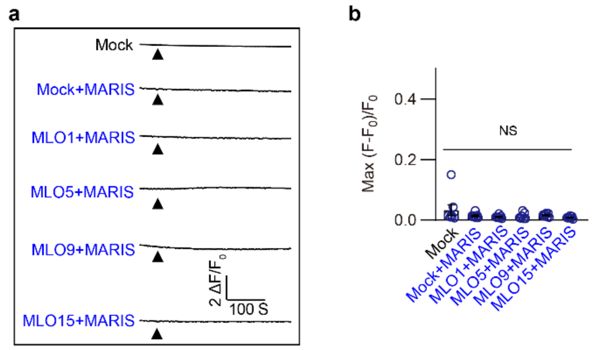

Supplementary information, Fig.2 Ca^{2+} imaging of COS7 cells expressing MLO1/5/9/15 and MARIS. a-b Representative cytosolic Ca^{2+} spiking curves (**a**) and statistical analysis of peak values (**b**) in COS7 cells co-expressing MARIS and each of the pollen tube MLOs. n =8 replicates, and ~ 60 cells were imaged in each duplicate. NS, not significant.
