# Supplementary material for: RALF signaling pathway activates MLO calcium channels to maintain pollen tube integrity
Source: Cell Res. 2023 Jan 2;33(1):71–9. doi: 10.1038/s41422-022-00754-3 (PMC9810639; doi:10.1038/s41422-022-00754-3)
Supplement: Supplementary file 8 — figS3 [file 41422_2022_754_MOESM8_ESM.pdf]

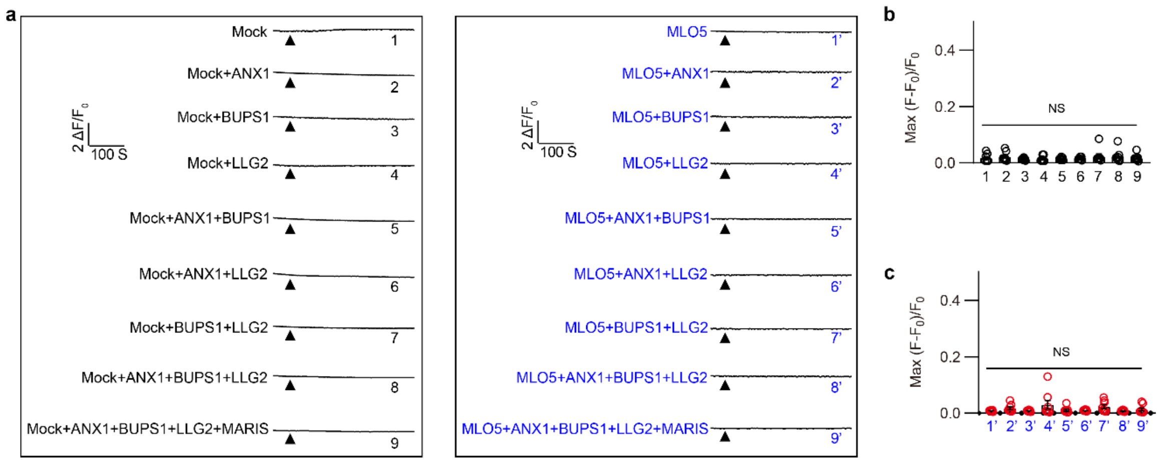

Supplementary information, Fig.3 MLO5 is not activated by RALF4/19 signaling components without the addition of RALF4 or 19. Representative cytosolic Ca^{2+} spiking curves (**a**) and statistical analysis of peak values (**b-c**) in COS7 cells co-expressing the MLO5 and RALF4/19 signaling components. n =8 replicates, and ~ 60 cells were imaged in each duplicate. NS, not significant.
