# Supplementary material for: RALF signaling pathway activates MLO calcium channels to maintain pollen tube integrity
Source: Cell Res. 2023 Jan 2;33(1):71–9. doi: 10.1038/s41422-022-00754-3 (PMC9810639; doi:10.1038/s41422-022-00754-3)
Supplement: Supplementary file 9 — figS4 [file 41422_2022_754_MOESM9_ESM.pdf]

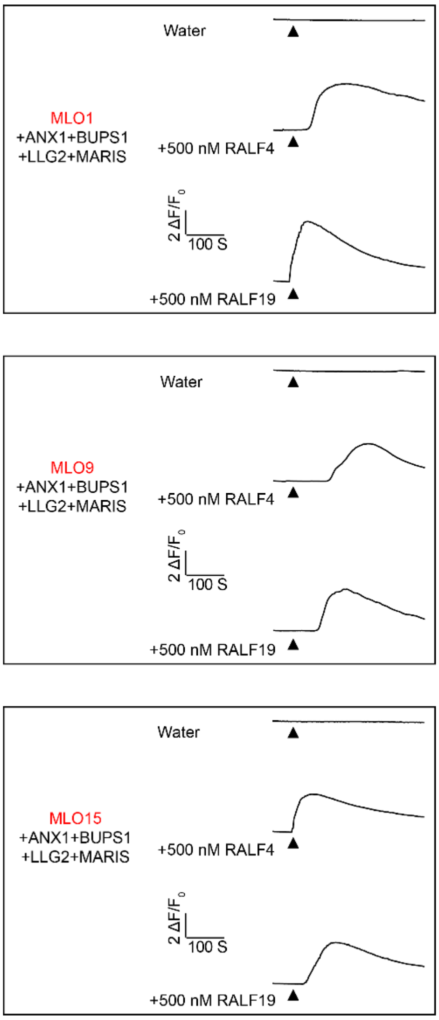

Supplementary information, Fig.4 Representative cytosolic Ca^{2+} spiking curves in COS7 cells co-expressing each of MLO1/9/15 and RALF4/19 signaling components upon addition of 500 nM RALF4 or RALF19 into the medium.
