# Supplementary material for: RALF signaling pathway activates MLO calcium channels to maintain pollen tube integrity
Source: Cell Res. 2023 Jan 2;33(1):71–9. doi: 10.1038/s41422-022-00754-3 (PMC9810639; doi:10.1038/s41422-022-00754-3)
Supplement: Supplementary file 10 — Table S1 [file 41422_2022_754_MOESM10_ESM.pdf]

Parent, Male X female	Expected	Observed	Transmission, %	P values
mlo5(+/-) *mlo9*(-/-) X *ms1*	160:160	140:180	77.8	0.0253
mlo5(-/-) *mlo9*(+/-) X *ms1*	201:201	180:222	81	0.0362
mlo1(-/-) *mlo5*(+/-) *mlo9*(-/-) X *ms1*	48:48	30:66	45.4	0.0002
mlo1(-/-) *mlo5*(-/-) *mlo9*(+/-) X *ms1*	96:96	68:124	54.8	<0.0001

Supplementary information, Table S1 T-DNA Segregation analysis reveals more severe defect in pollen transmission associated with *mlo1 mlo5 mlo9* triple mutant. *male sterility 1* (*ms1*) was used as female donor. Expected values are based on Mendelian segregation. *P* values are determined by the Chi-Squared test.
