# Supplementary material for: RALF signaling pathway activates MLO calcium channels to maintain pollen tube integrity
Source: Cell Res. 2023 Jan 2;33(1):71–9. doi: 10.1038/s41422-022-00754-3 (PMC9810639; doi:10.1038/s41422-022-00754-3)
Supplement: Supplementary file 11 — video description [file 41422_2022_754_MOESM11_ESM.pdf]

Supplementary information, Video S1

The application of pollen germination medium could not induce pollen tube Ca^{2+} elevation.

Supplementary information, Video S2

The application of 500 nM RALF4 triggered an exaggerated pollen tube Ca^{2+} elevation.

Supplementary information, Video S3

In wild type pollen grains, a Ca^{2+} spike appeared in the aperture area of pollen grain before tube protrusion, followed by a tip-focused calcium signal in the elongating pollen tube.

Supplementary information, Video S4

In *ralf4 ralf19* pollen grains, no Ca^{2+} elevation was detected before the pollen grain collapsed.

Supplementary information, Video S5

In the pollen grain overexpressing $MARIS^{R240C}$, Ca^{2+} flooded almost the entire pollen grain during germination, instead of forming a polarized Ca^{2+} signal at the aperture
